# Dictionary learning LASSO for feature selection with application to hepatocellular carcinoma grading using contrast enhanced magnetic resonance imaging

**DOI:** 10.3389/fonc.2023.1123493

**Published:** 2023-04-06

**Authors:** Lei Lei, Li-Xin Du, Ying-Long He, Jian-Peng Yuan, Pan Wang, Bao-Lin Ye, Cong Wang, ZuJun Hou

**Affiliations:** ^1^ College of Information Science and Engineering, Jiaxing University, Jiaxing, China; ^2^ Medical Imaging Department, Shenzhen Longhua District Central Hospital, Shenzhen, China; ^3^ School of Mechanical Engineering Sciences, University of Surrey, Guildford, United Kingdom; ^4^ Department of Radiology, The Seventh Affiliated Hospital, Sun Yat-sen University, Shenzhen, China; ^5^ Jiangsu Key Laboratory of Medical Optics, Suzhou Institute of Biomedical Engineering and Technology, Chinese Academy of Sciences, Suzhou, China

**Keywords:** hepatocellular carcinoma (HCC), radiomics, feature selection, magnetic resonance imaging (MRI), least absolute shrinkage and selection operator (LASSO) dictionary learning

## Abstract

**Introduction:**

The successful use of machine learning (ML) for medical diagnostic purposes has prompted myriad applications in cancer image analysis. Particularly for hepatocellular carcinoma (HCC) grading, there has been a surge of interest in ML-based selection of the discriminative features from high-dimensional magnetic resonance imaging (MRI) radiomics data. As one of the most commonly used ML-based selection methods, the least absolute shrinkage and selection operator (LASSO) has high discriminative power of the essential feature based on linear representation between input features and output labels. However, most LASSO methods directly explore the original training data rather than effectively exploiting the most informative features of radiomics data for HCC grading. To overcome this limitation, this study marks the first attempt to propose a feature selection method based on LASSO with dictionary learning, where a dictionary is learned from the training features, using the Fisher ratio to maximize the discriminative information in the feature.

**Methods:**

This study proposes a LASSO method with dictionary learning to ensure the accuracy and discrimination of feature selection. Specifically, based on the Fisher ratio score, each radiomic feature is classified into two groups: the high-information and the low-information group. Then, a dictionary is learned through an optimal mapping matrix to enhance the high-information part and suppress the low discriminative information for the task of HCC grading. Finally, we select the most discrimination features according to the LASSO coefficients based on the learned dictionary.

**Results and discussion:**

The experimental results based on two classifiers (KNN and SVM) showed that the proposed method yielded accuracy gains, compared favorably with another 5 state-of-the-practice feature selection methods.

## 1 Introduction

With an estimated incidence of >1 million cases by 2025, liver cancer remains a global health challenge (1). Hepatocellular carcinoma (HCC) is the most common form of liver cancer and accounts for 90% of cases, most of which occur in the setting of chronic liver disease (2). In clinical practice, different stages (referring to how far a cancer tumor has grown and spread) of HCC have different surgical cure rates, recurrence rates, and survival rates, and require different treatment approaches (3–5). Otherwise, inappropriate treatment makes HCC more prone to relapse and metastasize, associated with a poor prognosis (3, 4). Histopathologic grading describes how abnormal the cancer cells or tissue look under a microscope, which helps to predict how quickly cancer will grow and spread. Accurate visual assessment of HCC grading is essential for clinical decision-making, treatment regimen optimization, and prognostic prediction (5).

Current approaches to predict HCC grading include tumor biopsy (6), postoperative histopathological examination (7), ultrasound (8), computed tomography (CT) (9) and magnetic resonance imaging (MRI) (10, 11), etc. Among them, MRI is the most popular examination method for HCC grading due to its noninvasive, good soft-tissue resolution, and absence of radiation exposure.

Historically, in radiology practice, magnetic resonance imaging (MRI)-based HCC grading requires visual inspection by a radiologist. Such assessment, however, is often based on education and experience and can be, at times, subjective and time-consuming. Moreover, the visual judgment of MRI image sequences by radiologists only provides limited information on tumor heterogeneity, e.g., tumor location, size, peritumoral edema, morphology, and borders (12). In contrast to such qualitative reasoning, machine learning (ML) excels in identifying intricate patterns in imaging data and can automatically provide a quantitative evaluation. More accurate and reproducible radiology assessments can then be made when ML is incorporated into the clinical process as a tool to support radiologists (13).

In literature, several ML models have been proposed to automate HCC grading, and the results are promising (14–17). In those models, one of the most critical steps is feature extraction. In previous studies, many feature extraction methods were developed, such as texture features (17), shape features (18), radiomics features (15), deep learning features (19), and multi-fractal features (20). Although deep learning features can leverage neural networks (NNs) and demonstrate an exceptional ability to learn high-level features from data in an incremental manner, they are not widely used as a replacement for experienced radiologists. Instead, they have the potential to improve the accuracy and efficiency of diagnostic processes in clinical practice. This is due to the following reasons:

- the shallow NNs cannot effectively extract complex features;
- the deep NNs may suffer from the vanishing gradient problem;
- the internal mechanisms and the resulting features are often not explainable.

By contrast, radiomics can use a large number of quantitative image features to characterize tumor heterogeneity, providing a better understanding of cancer imaging data for clinical decision-making. Several studies have reported the successful applications of radiomics in HCC grading (15, 21, 22).

However, radiomic features usually have thousands of variables, leading to computational burden and the overfitting problem. Recognizing this deficiency, an emerging solution is to select the most discriminative features as input data for HCC grading. The existing methods of feature selection are generally classified into three categories:

- Filter methods, including statistical (such as descriptive and statistical dependency (DSD) method) (23), mutual information (such as artificial variables and mutual information (AVMI) method) (24), and reliefF (25), etc. Although these methods are simple and fast, their selection process is independent (i.e., not considering the interaction between the chosen features and the grading model), which compromises the grading accuracy. To overcome this limitation, some researchers combine various methods (26). For example, Qi et al. (26) take both inter- and intra-factors into account and combine the variance filter, t-test, and correlation coefficient on three MR to sequentially ensure the greatest diagnostic value. This model yields improved performance, but it is too complex to be accepted clinically.
- Wrapped methods (27, 28). The selection features based on the wrapper are evaluated and selected by an ML model. Although the wrapper methods outperform the filter-based equivalents, their learning process is data-hungry and time-consuming, especially for the high-dimension data set (29).
- Embedded methods, e.g., random forest(RF) based (30) and least absolute shrinkage and selection operator (LASSO) based (15). These methods consider all features as a whole and take the learning performance into account. These approaches have the advantages of both filter and wrapper methods and are widely recommended by researchers.

Recently, a growing body of literature has investigated embedded feature selection methods using LASSO, achieving desirable performance in different fields (31–34). Wang et al. (33) combined Chi-square and LASSO (Chi+LASSO) for selecting radiomics features of HCC MRI data, where chi-square and LASSO were used for univariate selection and multivariate selection, respectively.

However, previous studies on LASSO based feature selection approaches treat radiomic features equally for HCC grading. Since these features can easily be affected by many factors, e.g., hardware configuration, acquisition, data postprocessing, software implementation, and noise, the meaningful features may not be evaluated correctly, which may influence the weights of the features, thus, deteriorate the performance of the grading model. To overcome this limitation, this study proposes a LASSO method with dictionary learning to ensure the accuracy and discrimination of feature selection. Specifically, based on the Fisher ratio score, each radiomic feature is classified into two groups: the high-information and the low-information group. Then, a dictionary is learned through an optimal mapping matrix to enhance the high-information part and suppress the low discriminative information for the task of HCC grading. Finally, we select the most discrimination features according to the LASSO coefficients based on the learned dictionary. Experimental results indicate that the developed feature selection method can select the most informative data from the high-dimensional radiomic features, and lead to enhanced performance in subsequent HCC grading.

## 2 The LASSO model for features selection

Given a dictionary *X* ϵ *R^n×k^* consists of the radiomic features of training data, where *k* is the dimension of the radiomic features, *n* is the number of training data. The label *y* ϵ *R^n×1^* can be described as


(1) y=Xα+e,

where *α* is the weight vector to be estimated, e is the error vector whose entries are assumed to be small. If we can find *α* with a few nonzero entries such that *Xα* ≈ *y*, then the sparse vector can provide the predictive relationships which generalize well to the test data. In short, the greater the value of the element of *α*, the more effective the element for HCC grading. Therefore, based on the value of *α*, we can select the first τ radiomic features as the grading features, where τ is a constant. The value of *α* can be estimated by solving the LASSO model:


(2) $$\hat{\alpha }=\underset{\alpha }{\arg\;\min\;}\|X\alpha -y{\|}_{2}^{2}+\mu \|\alpha {\|}_{1}$$

It has been reported that the choice of X is essential (35). In previous studies, the dictionary is largely directly constructed from the radiomic features of original training data. This work proposes a learning method to define an adaptive dictionary based on the contribution of both the radiomic features of training data and the coefficient vector *α*, as described in the next section.

## 3 The proposed features selection method

### 3.1 Dictionary learning

The proposed dictionary learning method is based on the radiomic features of original data. We define the initial dictionary as $D_{0}=\left[d_{1},d_{2},\ldots ,d_{n}\right]\in R^{k\times n}$ , where $d_{i},i=1,2,\ldots ,n$ is the radiomic feature of the *ith* sample. We then decompose each feature $d_{i}$ into two parts, part $d_{i}^{h}$ , which is more effective in HCC grading, and part $d_{i}^{l}$ , which is less effective in HCC grading. Furthermore, the dictionary $D_{0}$ can be divided into two parts as,


(3) $$D_{0}=D^{h}+D^{l}$$

Here, part $D^{h}$ includes more informative components (called the high information part, HIP) and the other part $D^{l}$ contains less informative components (called the low information part, LIP). To effectively exploit the useful information in both $D^{h}$ and $D^{l}$ , a projection matrix P is designed to map the initial dictionary into a new one, such that the energy of $D^{h}$ would be effectively preserved, while that of $D^{l}$ would be suppressed.

Defined ${\bar{d}}_{c}^{h}$ , ${\bar{d}}_{c}^{l}$ , ${\bar{d}}^{h}$ are the mean vector of all the features in $D^{h}$ belonging to *cth* grade, all the features in $D^{l}$ belonging to *cth* grade, and all the features in $D^{h}$ , respectively. ${\bar{d}}_{i,c}^{h}=d_{i}-{\bar{d}}_{c}^{h},\;{\bar{d}}_{i,c}^{l}=d_{i}-{\bar{d}}_{c}^{l},\;{\bar{d}}^{c,h}={\bar{d}}_{c}^{h}-{\bar{d}}^{h}$ are the centralized image vectors. To make the selected features effective in HCC grading, it is necessary to take into account both between-class and within-class variation in the design of project matrix P . We can construct the between-class average projection energy of HIP as:


(4) $$\begin{array}{l} E_{B}^{h}=\sum_{c=1}^{C}\|P{\bar{d}}^{c,h}{\|}_{2}^{2}\\ \;=\sum_{c=1}^{C}tr\left\{\left(P{\bar{d}}^{c,h}\right){\left(P{\bar{d}}^{c,h}\right)}^{T}\right\}\\ \;=tr\left\{P\left(\sum_{c=1}^{C}{\bar{d}}^{c,h}{\left({\bar{d}}^{c,h}\right)}^{T}\right)P^{T}\right\}\\ \;=tr\left(PS_{B}^{h}P^{T}\right) \end{array}$$

where tr is the matrix trace operator, $S_{B}^{h}=\sum_{c=1}^{C}\left({\bar{d}}_{c}^{h}-{\bar{d}}^{h}\right){\left({\bar{d}}_{c}^{h}-{\bar{d}}^{h}\right)}^{T}$ is the scatter matrix of $D^{h}$ , *C* is the number of grades, in this study we set it as 2. The average within-class projection energy of HIP is defined as,


(5) $$\begin{array}{l} E_{w}^{h}=\sum_{c=1}^{c}\sum_{d_{i}\in D_{c}}∥P{\bar{d}}_{i,c}^{h}{∥}_{2}^{2}\\ \;=\sum_{c=1}^{c}\sum_{d_{i}\in D_{c}}tr\left\{\left(P{\bar{d}}_{i,c}^{h}\right){\left(P{\bar{d}}_{i,c}^{h}\right)}^{T}\right\}\\ \;=tr\left\{P\left(\sum_{c=1}^{C}\sum_{d_{i}\in D_{c}}{\bar{d}}_{i,c}^{h}{\left({\bar{d}}_{i,c}^{h}\right)}^{T}\right)P^{T}\right\}\\ \;=tr\left(PS_{w}^{h}P^{T}\right), \end{array}$$

where $D_{c}$ is the set of radiomic features of the cth class, $S_{W}^{h}=\sum_{c=1}^{C}\sum_{d_{i}\in D_{c}}\left(d_{i}-{\bar{d}}_{c}^{h}\right){\left(d_{i}-{\bar{d}}_{c}^{h}\right)}^{T}$ . Similarly, we can get $E_{W}^{l}=tr\left(PS_{W}^{l}P^{T}\right)$ , where $S_{W}^{l}=\sum_{c=1}^{C}\sum_{d_{i}\in D_{c}}\left(d_{i}-{\bar{d}}_{c}^{l}\right){\left(d_{i}-{\bar{d}}_{c}^{l}\right)}^{T}$ .

The projection matrix P is designed to facilitate HCC grading. To that end, we need to maximize the between-class average projection energy $E_{B}^{h}$ and minimize the within-class average projection energy $E_{W}^{h}$ and $E_{W}^{l}$ , by solving the following optimization problem,


(6) $$\begin{array}{l} \hat{P}=\arg\;\underset{P}{\max}\frac{tr\left\{PS_{B}^{h}P^{T}\right\}}{\beta \cdot tr\left\{PS_{W}^{h}P^{T}\right\}+\left(1-\beta \right)tr\left\{PS_{W}^{l}P^{T}\right\}}\\ \;=\arg\;\underset{P}{\max}\frac{tr\left\{PS_{B}^{h}P^{T}\right\}}{tr\left\{P\left(\beta \cdot S_{W}^{h}+\left(1-\beta \right)S_{W}^{l}\right)P^{T}\right\}} \end{array}$$

where scalar β is used to balance the within-class energy of HIP and LIP. It is noted that two important aspects can affect the effectiveness of the above dictionary learning process, namely, the grouping of atoms $d_{i}$ to obtain the decomposed D in Eq. (3) and the solution of the optimization problem in Eq. (6), which will be addressed in Subsections 3.2 and 3.3, respectively.

### 3.2 Feature grouping

In this retrospective study, the grading label of $y_{i}$ is available. In this case, we introduce the Fisher ratio to group the features. If the feature has a bigger Fisher ratio, this feature is more discriminative in grading the lesions. Based on this heuristic, we can group the features into a more discriminative group and a less discriminative group. For each feature vector $d_{i}$ , we represent it by the initial dictionary $D_{0}$ :


(7) $$d_{i}\approx D_{0}\alpha =\alpha_{1}\cdot d_{1}+\alpha_{2}\cdot d_{2}+\ldots +\alpha_{n}\cdot d_{n}$$

where α is obtained *via* the LASSO. Let $z_{i}=\alpha_{i}\cdot d_{i}$ , the Fisher ratio $f_{i}$ of feature images $d_{i}$ is


(8) $$f_{i}=\frac{\sum_{c=1}^{C}{\left(\bar{z}-{\bar{z}}_{c}\right)}^{2}}{\sum_{c=1}^{C}\frac{1}{n_{c}}\sum_{d_{i}\in D_{c}}{\left(z_{i}-{\bar{z}}_{c}\right)}^{2}},$$

where $n_{c}$ is the number of features belonging to the cth grade, $\bar{z}$ is the mean vector of all the $z_{i}$ , ${\bar{z}}_{c}$ is the mean vector of $z_{i}$ that belongs to grade c . To this end, we re-order the $z_{i}$ according to the $f_{i}$ in descending order, those features which have larger $f_{i}$ are added up for the HIP $D^{h}$ , and the remaining features are added up for the LIP $D^{l}$ . For the convenience of expression, we suppose that vectors $\left\{z_{1},z_{2},\ldots ,z_{k}\right\}$ fall into the HIP and vectors $\left\{z_{k+1},z_{k+2},\ldots ,z_{n}\right\}$ fall into the LIP. Then we can define the high information part $d_{i}^{h}$ and low information part $d_{i}^{l}$ as


(9) $$\begin{array}{l} d_{i}^{h}=z_{1}+z_{2}+\ldots +z_{k},\\ d_{i}^{h}=z_{k+1}+z_{k+2}+\ldots +z_{n}. \end{array}$$

Then, each feature of the training lesion can be written as $d_{i}=d_{i}^{h}+d_{i}^{l}$ , and we have $D_{0}=D^{h}+D^{l}$ .

### 3.3 Solve the optimization problem

To solve the optimization problem in Eq. (6), let $S^{a}=S_{B}^{h}$ and $S^{b}=\beta \cdot S_{W}^{h}+\left(1-\beta \right)S_{W}^{l}$ . The matrix P is split into n vectors as $p_{1},p_{2},\ldots ,p_{n}$ , then we have,


(10) $$\begin{array}{l} tr(PS^{a}P^{T})\\ \;=tr\left(\left[\begin{array}{c} p_{1}\\ p_{2}\\ \vdots \;\\ p_{n} \end{array}\right]S^{a}[p_{1}^{T}\;p_{2}^{T}\;\cdots \;p_{n}^{T}]\right)\\ \;=tr\left(\left[\begin{array}{ccc} p_{1}S^{a}p_{1}^{T} & p_{1}S^{a}p_{2}^{T}\cdots & p_{1}S^{a}p_{n}^{T}\\ p_{2}S^{a}p_{1}^{T} & p_{2}S^{a}p_{2}^{T}\cdots & p_{2}S^{a}p_{n}^{T}\\ \vdots \; & \vdots & \;\vdots \\ p_{n}S^{a}p_{1}^{T} & p_{n}S^{a}p_{2}^{T}\cdots & p_{n}S^{a}p_{n}^{T} \end{array}\right]\right)\\ \;=\sum_{i=1}^{n}p_{i}S^{a}p_{i}^{T}. \end{array}$$

In the same way, we have


(11) $$tr\left(PS^{b}P^{T}\right)=\sum_{i=1}^{n}p_{i}S^{b}p_{i}^{T}.$$

Then, by substituting Eqs. (10)-(11) into Eq. (6), we have,

$\frac{tr\left(PS^{a}P^{T}\right)}{tr\left(PS^{b}P^{T}\right)}=\frac{\sum_{i=1}^{n}p_{i}S^{a}p_{i}^{T}}{\sum_{i=1}^{n}p_{i}S^{b}p_{i}^{T}}=\frac{\sum_{i=1}^{n}u_{i}}{\sum_{i=1}^{n}v_{i}}\leq \frac{\sum_{i=1}^{n}\left(u_{i}+\frac{u_{i}}{v_{i}}\sum_{j\neq i}v_{j}\right)}{\sum_{i=1}^{n}v_{i}}$


(12) $$=\frac{\sum_{i=1}^{n}\frac{u_{i}}{v_{i}}\sum_{i=1}^{n}v_{i}}{\sum_{i=1}^{n}v_{i}}=\sum_{i=1}^{n}\frac{u_{i}}{v_{i}}=\sum_{i=1}^{n}\frac{p_{i}S^{a}p_{i}^{T}}{p_{i}S^{b}p_{i}^{T}},$$

where $u_{i}$ and $v_{i}$ are defined as $u_{i}=p_{i}S^{a}p_{i}^{T}$ and $v_{i}=p_{i}S^{b}p_{i}^{T}$ , respectively. Due to the fact that both $S^{a}$ and $S^{b}$ are positive definite matrices, we have $u_{i}\geq 0$ and $v_{i}\geq 0$ . For the two matrices $S^{a}$ and $S^{b}$ , their generalized eigenvalues and eigenvectors are defined as $\lambda_{i}$ ( i=1,2,…,n ) and $q_{i}$ ( i=1,2,…,n ), respectively, leading to,


(13) $$\max\;\frac{q_{i}S^{a}q_{i}^{T}}{q_{i}S^{b}q_{i}^{T}}=\lambda_{i}.$$

Apparently, the desired P is composed of the generalized eigenvectors of $S^{a}q_{i}=\lambda_{i}S^{b}q_{i}$ , corresponding to the n largest eigenvalues, i.e. $P=\left[q_{1},q_{2},\cdots ,q_{n}\right]$ .

## 4 Materials and workflow for HCC grading

**Figure 1:**
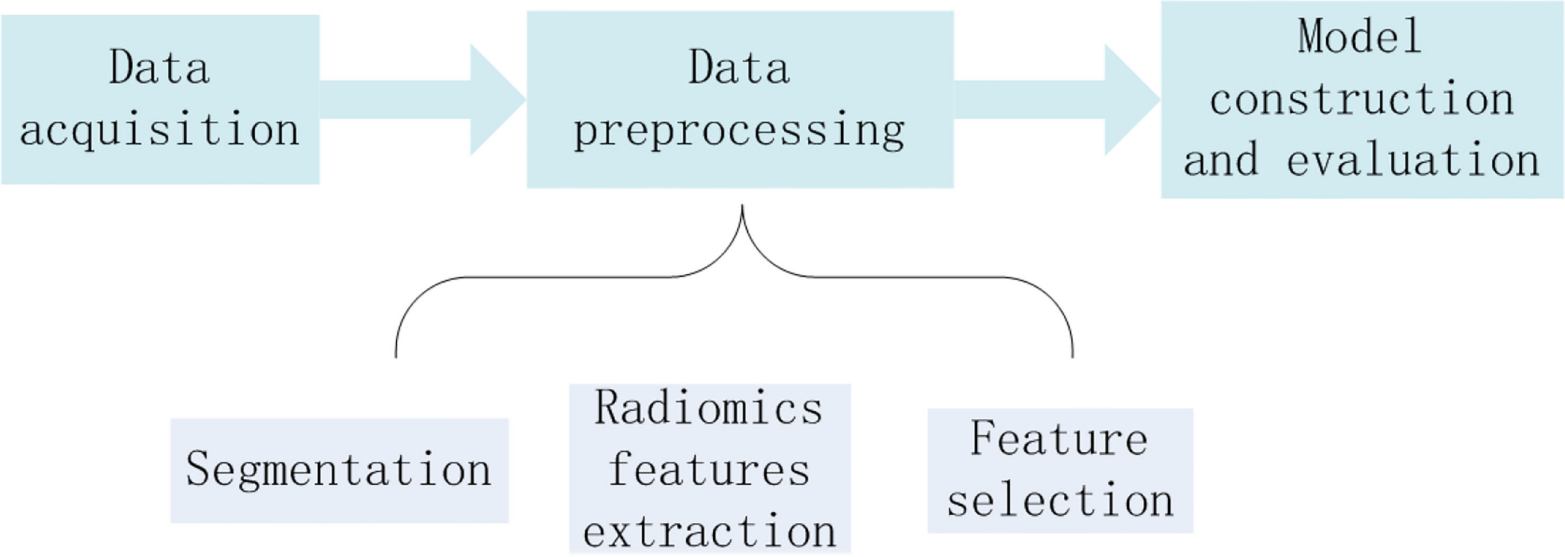
The workflow of HCC grading.

The workflow of this study was illustrated in **Figure 1**, as detailed in the following.

### 4.1 Patient data

This retrospective study was approved by the institutional review board and patient informed consent was waived. MRI data of 462 patients examined from June 2016 to June 2021 at Sun Yat-sen University Cancer Hospital and pathologically confirmed as HCC were reviewed. Among them, 367 patients (284 males and 83 females, mean age 49.7 years, and age range 18-81 years) were included in the final analysis. The inclusion criteria were as follows:

1. A complete clinical reports and be pathologically confirmed as HCC;
2. Dynamic contrast enhanced MRI (DCE-MRI) examination within seven days before surgery;
3. Staging results;
4. No history of other types of tumor.

We identified a total of 599 lesions from these HCC patients based on the liver imaging reporting and data system (LI-RADS) v2018 criteria [9]. The detailed characteristics and statistics for these patients were shown in **Table 1**.

**Table 1:** Characteristics of included HCC patients.

Item	Total	Training	Test
Age			
Range	18-81	18-81	18-76
Mean	49	49	49
Gender			
Male	284	200	84
Female	83	59	24
Lesion			
Male	465	321	144
Female	134	98	36
Stage			
LR-1	145	106	39
LR-2	172	108	64
LR-3	37	28	9
LR-4	54	39	15
LR-5	191	138	53

### 4.2 Data acquisition

All examinations had been performed on a 3.0T MRI scanner (Skyra, Siemens, Germany) with a sixteen-channel phase array coil that covered the entire liver. Routine MRI protocols included a respiratory-triggered fat-suppressed T1-weighted dual-echo sequence (DE-T1WI), a respiratory-triggered fat-suppressed T2-weighted fast spin-echo sequence (FSE-T2WI), and a diffusion-weighted sequence (DWI). The scanning parameters of different MRI sequences are shown in **Table 2**.

**Table 2:** Scan parameters of different routine MRI sequences.

sequence parameter	DE-T1WI	FSE-T2WI	DWI
TR(ms)	4.5	3000	3500
TE(ms)	1.29/2.52	84	59
Slice thickness(mm)	3	5	5
Gap of slice(mm)	0.6	1	1
FOV(mm × mm)	380 × 340	380 × 380	380 × 340
Matrix	195 × 320	320 × 320	108 × 128
b values	—	—	0,400,1000*s*/*mm* ^2^

TR-repetition time; TE-echo time; FOV-field of view.

Contrast agents (GD-EOB-DTPA, Primovist, Bayer) were administered with an injection rate of 2 ml/s and gadolinium dose of 0.1 mmol/kg body weight, followed by a 20 ml normal saline flush. The post-contrast scan was performed at four different phases: arterial phase (30 s after contrast injection), portal venous phase (60 s), transitional phase (180 s) and hepatobiliary phase (20 mins), respectively. The post-contrast scan sequence was T1-weighted 3D gradient echo sequence with fat saturation and volumetric interpolated breath-hold examination with the parameters of slice thickness (6 mm), TR (3.12 ms), TE (1.51 ms), matrix (290×290), and flip angle (10°).

### 4.3 Manual segmentation and grading

HCC lesions were manually segmented by two radiologists (LD and PW) with thirty and nine years of experience, respectively. For each case, one radiologist manually delineated the maximum extent of the visible lesion using ITK-SNAP 3.8.0 without prior knowledge of the histopathological results. The other radiologist reviewed the segmentation result independently to sure the accuracy of the segmentation. In addition, for the purpose of quantitative comparisons the segmentation agreements, three assessment metrics including dice coefficient (DC) (36), global consistency error (GCE) (37) and probabilistic rand index (PRI) (38, 39), are introduced to evaluate the segmentation results. The values of DC, GCE and PRI are 0.873, 0.052 and 0.792, respectively. Note that all the values were calculated based on the region of interest rather than the full image. By segmenting the data volumes, a total of 599 lesions were obtained. The min, max, mean, and median values of tumor volume are 29 voxels, 800842 voxels, 16346 voxels and 1588 voxels, respectively.

**Figure 2:**
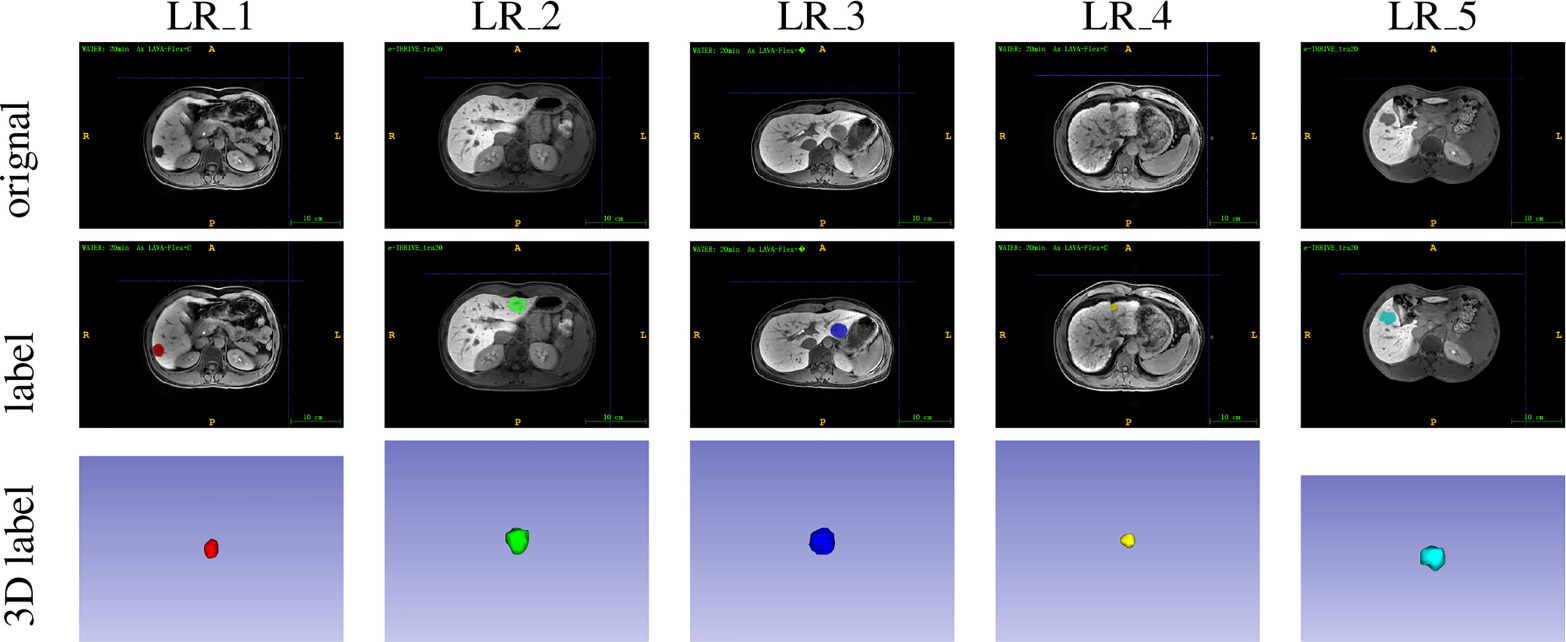
The segmentation and grading results of representative images.

**Figure 3:**
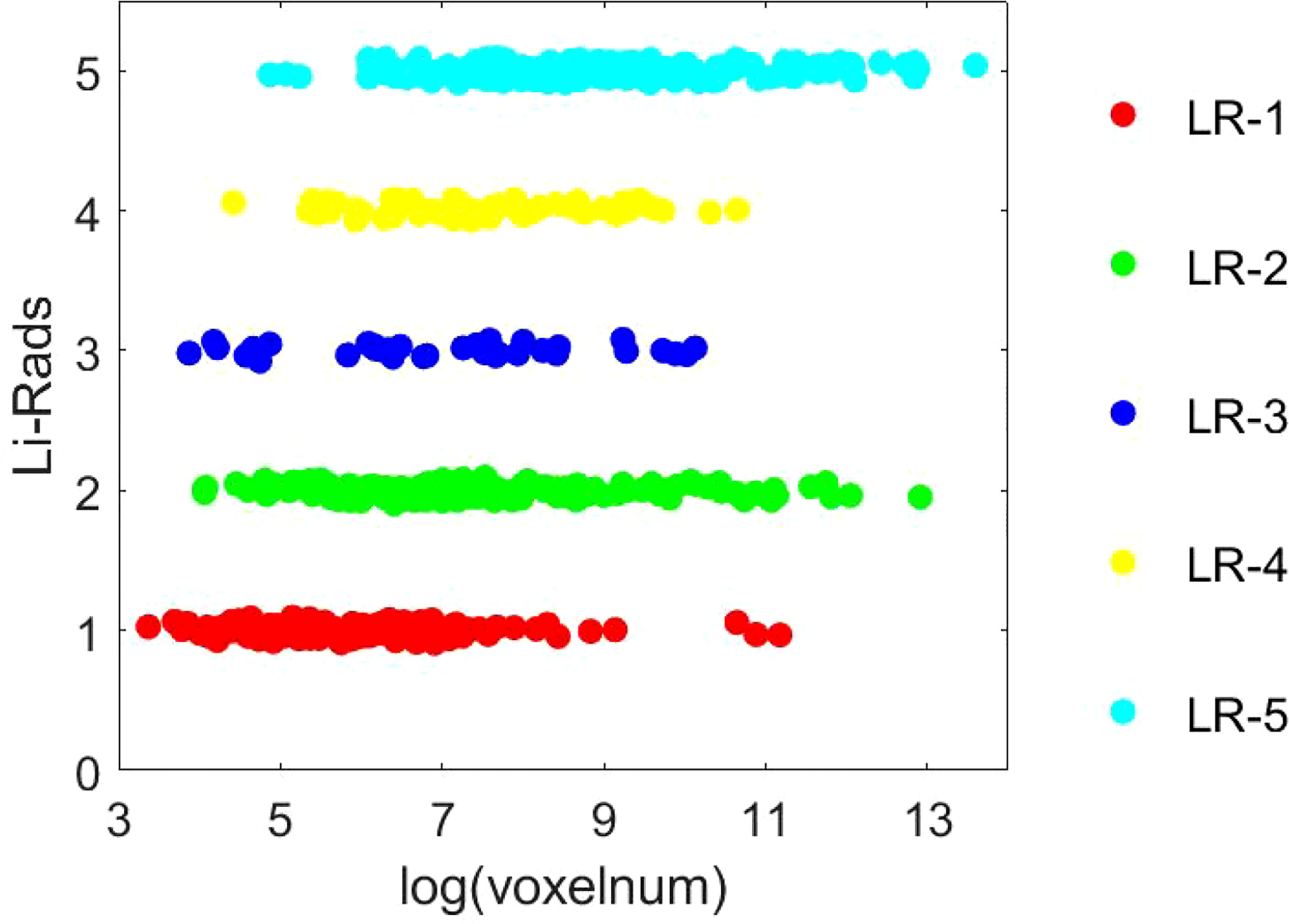
The distribution of LI-RADS results.

After segmentation, these two radiologists independently assigned a grading label for each lesion according to the LI-RADS v2018 criteria (40). Disagreements regarding the LI-RADS categorization were resolved by consensus with a senior abdominal radiologist (JY) with over 32 years of 204 liver imaging experience. Based on the LI-RADS categories, the lesions are classified as low-grade HCC (LR-1 and LR-2) and high-grade HCC (LR-3, LR-4 and LR-5) in this study. Representative images from 206 HCC patients and the corresponding segmentation and grading results were presented in **Figure 2**, and the 207 LI-RADS distribution of all lesion data was shown in the **Figure 3**.

### 4.4 Feature extraction and selection

Feature extraction was performed by using an open-source Python package (Pyradiomics V2.1.2) for each lesion. The extracted features were divided into the following seven categories:

- first-order statistical properties;
- gray level co-occurrence matrix (GLCM);
- gray level dependence matrix (GLDM);
- gray level run length matrix (GLRLM);
- grar level size zone matrix (GLSZM);
- neighborhood gray tone difference matrix (NGTDM);
- 2D shape features.

Among them, the 2D shape features are calculated from the original images. The other six features are calculated based on the original images, Laplacian of Gaussian filtered images (with a kernel size of 1.5 mm and 2.5 mm) and wavelet-based images. A total of 1050 features were extracted for each lesion. Detailed information about the feature extraction method and filters can be found on the web here.

Note that before feature selection, the features were first normalized (Z-score normalization) to ensure a relatively uniform distribution of the image features.

**Figure 4:**
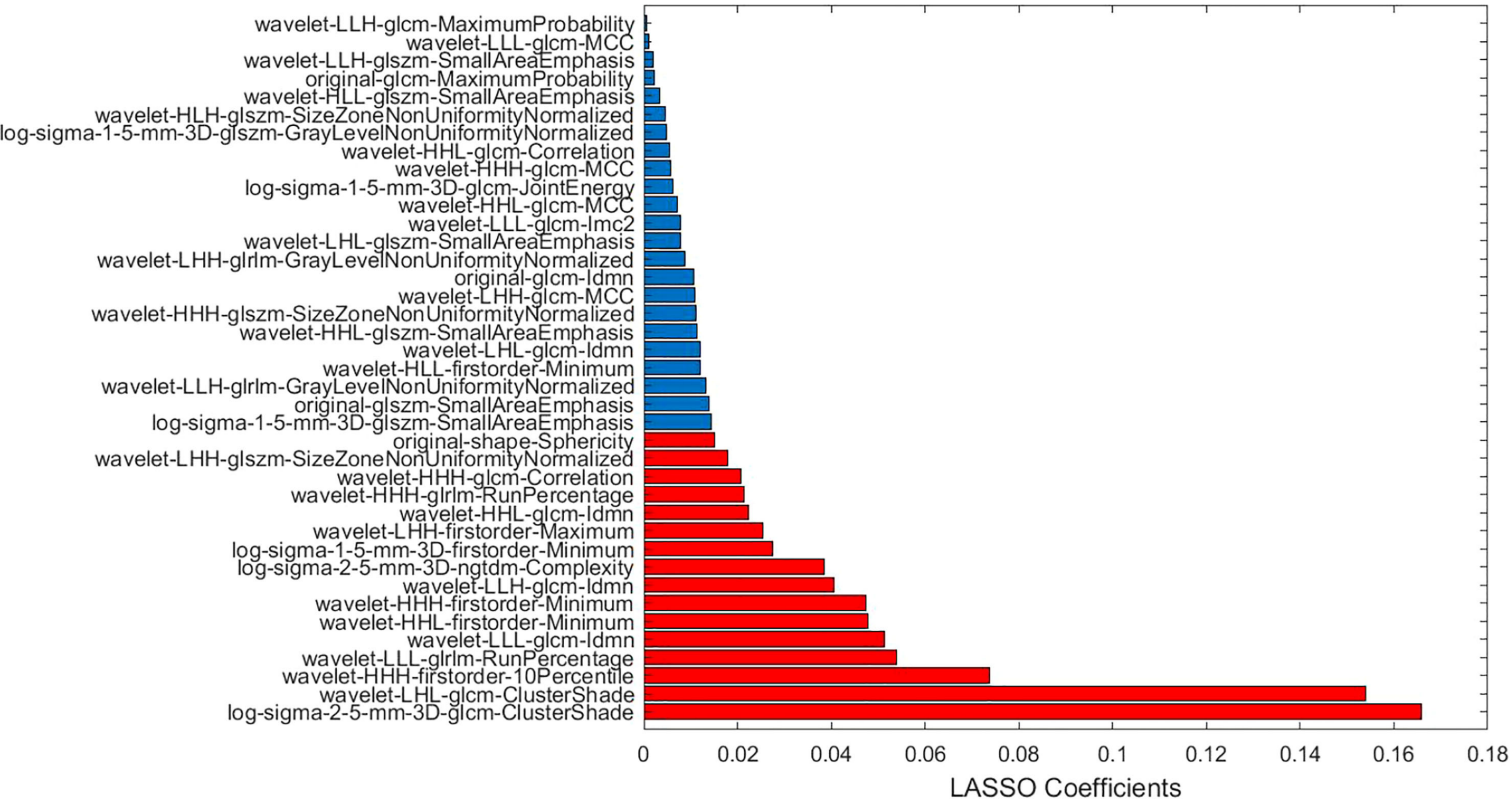
The non-zero coefficients in dl-LASSO and the selected features by dl-LASSO (red mark).

The proposed dl-LASSO for feature selection has been introduced in detail in the above Sections 2 and 3. The weights of all the non-zero features and the selected features were shown in **Figure 4**.

### 4.5 HCC grading

All the lesions were randomly divided into 7:3 partitions and were utilized as training and validation sets. The statistics for the training and test data were shown in **Table 1**.

Two machine learning classifiers were used for HCC grading in this study, i.e. support vector machine (SVM) (41) and K-nearest neighbor (KNN) (42). The operating environment of both classifiers is MATLAB 2021.

#### 4.5.1 Support vector machine (SVM)

As one of the most popular classifiers, the basic design philosophy of SVM is to maximize the classification boundaries and the hyper-plane (43). For training pairs $\left(d_{i},y_{i}\right),i=1,2,\ldots ,n$ , the SVM requires solving the following optimization problem.


(14) $$\begin{array}{l} \underset{\omega ,b}{\min}\omega^{T}\omega /2+C\sum_{i=1}^{n}\xi_{i}\circ s.t.\circ y_{i}\left(\omega^{T}\phi \left(d_{i}\right)+b\right)\geq 1-\xi_{i}, \end{array}$$

where $\xi_{i}$ is a non-negative relaxation variable, ϕ is a function that maps the vector $d_{i}$ into a higher dimensional space. Then SVM finds a linear separating hyperplane with the maximal margin in this higher dimensional space. Furthermore, we call $K\left(d_{i},d_{j}\right)=\phi \left(d_{i}\right)\phi \left(d_{j}\right)$ as the kernel function. In this study, radial basis function kernels have been selected, and 10-fold cross-validation was used.

#### 4.5.2 K-nearest neighbors (KNN)

In addition to SVM, we introduce another KNN classification approach to grade the HCC lesion. KNN is one of the simplest and most commonly used classification methods, it classifies the data according to the distance information of K nearest neighbors. For a test sample $d_{t}$ , calculates the Euclidean distance between it and the training data *d*_1_,*d*_2_,…,*d_n_*,


(15) $${Ed}_{i}=\sqrt{\sum_{i=1}^{n}{\left(d_{t}-d_{i}\right)}^{2}},$$

According to the calculated distance values, the category with the most occurrence of the KNN is the category of the test sample. In this study, the *K* is set as 7.

### 4.6 Evaluation

The effectiveness of HCC grading was evaluated based on the following performance indicators: recall, precision, F1-score, accuracy, and the area under curve (AUC) from the receiver operator characteristic (ROC) curve. The ROC curve was drawn according to the False Positive Rate and True Positive Rate. The calculation methods of these five indicators were shown in **Table 3**. All the experiments were performed on a workstation with a 28-core Intel (R) Xeon (R) Gold 5120 CPU (2.5 GHz) with 128 GB of RAM, Windows 10 operating system.

**Table 3:** Performance indexes used for the evaluation and comparison of the estimated model.

Index	Formula
Recall	$\frac{TP}{TP+FN}$
Precision	$\frac{TP}{TP+FP}$
F1-score	$\frac{2\left(Precision\times Recall\right)}{Precision+Recall}$
Accuracy	$\frac{TP+TN}{TP+TN+FP+FN}$
False Positive Rate	$\frac{FP}{FP+TN}$
Ture Positive Rate	$\frac{TP}{TP+FN}$

TP, True Positive; TN, True Negative; FP, False Positive; FN, False Negative.

## 5 Experiments and discussion

**Figure 5:**
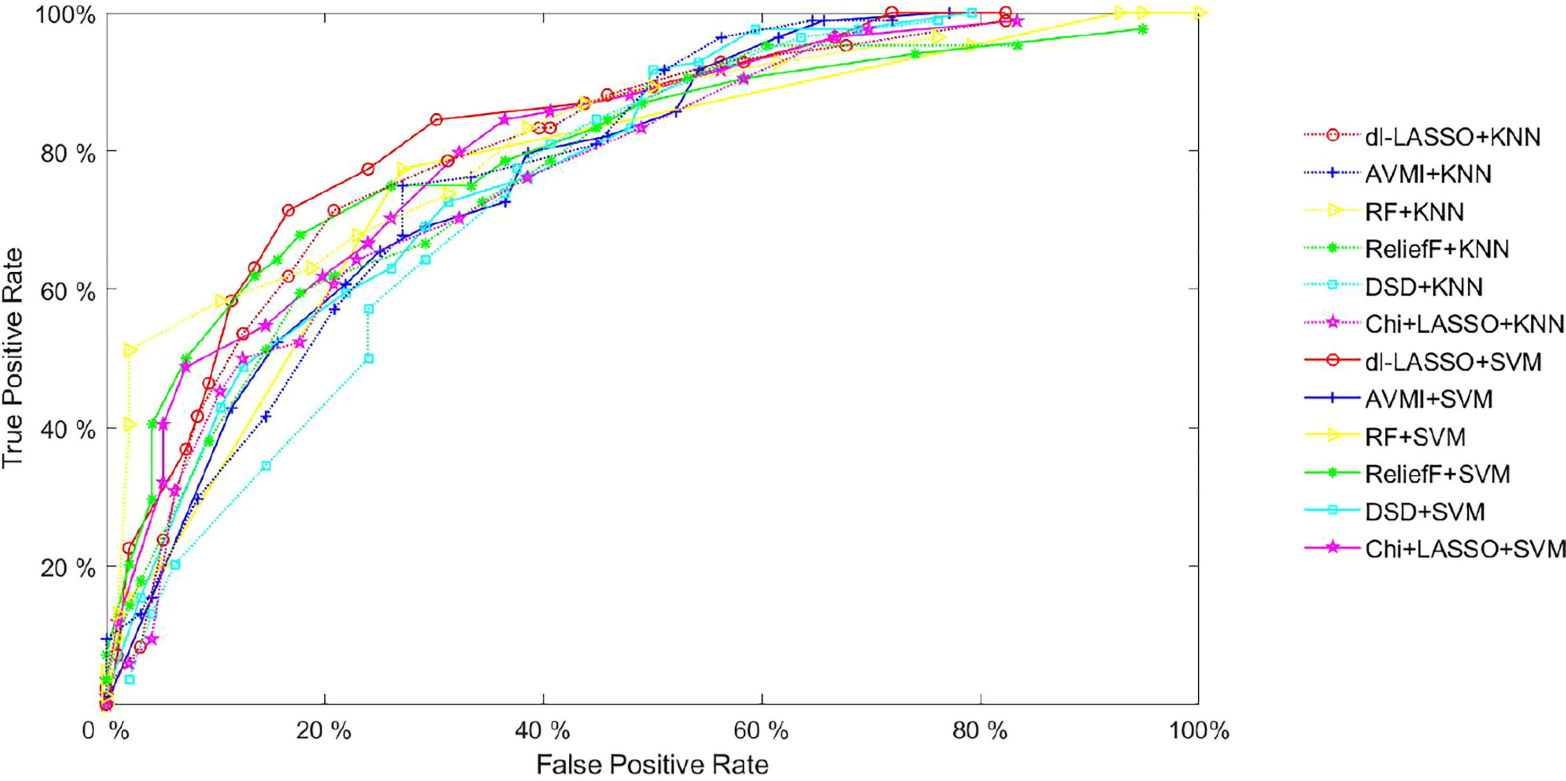
The ROC curves of KNN and SVM classifier.

To validate the effectiveness of the proposed HCC grading method based on dl-LASSO, we compared it with 5 other state-of-the-practice feature selection algorithms, including AVMI, RF, ReliefF, DSD, and Chi with LASSO. **Figure 5** compares ROC curves between the proposed feature selection method (i.e., dl-LASSO) and other methods (including AVMI, RF, ReliefF, DSD, Chi+LASSO) based on SVM and KNN classification models, respectively. A higher location of the ROC curve indicates a better grading quality. The figure shows that the curves of the proposed method are generally positioned higher than those of the other methods, although the improvements are not significantly apparent. In particular, for the curves derived from the KNN model, RF even outperforms the proposed model at low False Positive Rates (<20%). To conduct a more comprehensive comparison, additional index parameters are computed, which are presented in **Table 4** and discussed as follows.

**Table 4:** The ROC curves of KNN and SVM classifier.

Feature selection method	classification model	Recall	Precision	F1-Score	Accuracy	AUC
dl-LASSO	KNN	0.714	**0.750**	**0.732**	**0.756**	0.807
AVMI		**0.750**	0.708	0.728	0.739	0.779
RF		0.679	0.722	0.699	0.728	**0.822**
ReliefF		0.667	0.667	0.667	0.689	0.773
DSD		0.738	0.639	0.685	0.683	0.748
Chi+LASSO		0.643	0.711	0.675	0.711	0.771
dl-LASSO	SVM	**0.714**	**0.789**	**0.750**	**0.778**	**0.836**
AVMI		0.690	0.674	0.682	0.700	0.780
RF		0.202	0.773	0.321	0.600	0.804
ReliefF		0.679	0.770	0.722	0.756	0.811
DSD		0.690	0.674	0.682	0.700	0.782
Chi+LASSO		0.667	0.709	0.687	0.717	0.811

The bold values represent the best ones.

Recall denotes the accuracy in predicting positive cases. The recall values of the 6 feature selection methods based on KNN are 0.714, 0.750, 0.679, 0.667, 0.738, and 0.643, respectively. These outcomes indicate that AVMI achieves the highest accuracy in true lesion recognition, followed by the proposed method dl-LASSO. Although AVMI’s recall value (0.690) based on the SVM model is lower than that of the proposed method (0.714), AVMI still performs better than the other four methods in this regard. The results imply that AVMI’s integration of mutual information (MI) results in enhanced feature distinguishing ability. However, when the false lesion recognition is considered, AVMI is no longer a competitive method, for example, its precision value (0.708) is lower than that of the proposed method (0.750) and RF (0.722). This may be explained by the fact that AVMI only compares the MI value between the rearranged feature and the original one, without setting corresponding thresholds. Consequently, this may lead to the misidentification of benign tumors. More specifically, the lack of the thresholds in AVMI gives rise to the possibility of overweighting benign tumors with noise, which can result in their misclassification. As a result, this issue also affects the F1-score and accuracy metrics of AVMI, which are 0.728 and 0.739, respectively, based on KNN, and 0.682 and 0.700, respectively, based on SVM.

Precision measures the ability of selected features to identify benign tumors. The precision values of the proposed dl-LASSO method, based on KNN and SVM models, are 0.750 and 0.789, respectively, and both are superior to those of the other five methods. These findings indicate that the proposed method has a competitive edge in distinguishing benign tissue from noise information. This could be attributed to the consideration of the correlation within and between features in selection. It is worth noting an interesting aspect of the precision results, wherein the precision values of RF based on KNN and SVM models are 0.722 and 0.773, respectively, which are only slightly lower than those of the proposed method. Furthermore, RF’s AUC value based on KNN is relatively high (0.822). These imply that RF can be considered a competitive approach for feature selection. However, RF’s superior performance may not be entirely reliable, as suggested in the literature (44) and evidenced in **Table 4** by its low recall and F1-score values derived from the SVM model (0.202 and 0.321, respectively, both of which are the lowest). Therefore, additional research is necessary to further investigate the stability and reliability of the RF method.

Both the F1-score and accuracy metrics show that the proposed dl-LASSO method outperforms the others. The KNN- and SVM-based F1-score values are 0.732 and 0.750, respectively, while the accuracy values are 0.756 and 0.778, respectively - all of which are the highest. This underscores the superior ability of the proposed approach to accurately grade tumors using discriminative features. These findings provide strong evidence of LASSO’s effectiveness as a feature selection method with comprehensive recognition ability for lesions, benign tumors, and even noisy data. If unreliable values of RF are disregarded, the AUC results exhibit the same trend. AUC is a potent measure for grading, with higher values indicating greater grading accuracy. Based on KNN, the AUC values for six feature selection methods (the proposed dl-LASSO, AVMI, DSD, ReliefF, and Chi with LASSO) are 0.807, 0.779, 0.773, 0.748, and 0.771, respectively. Based on SVM, these values are 0.836, 0.780, 0.811, 0.782, and 0.811, respectively. These results suggest that the proposed method produces the best grading results.

**Table 4** also suggests that DSD’s performance is not outstanding, regardless of the indicator used. The KNN-based DSD yields values of Recall, Precision, F1-Score, Accuracy, and AUC at 0.738, 0.639, 0.685, 0.683, and 0.748, respectively. Based on SVM, these values are 0.690, 0.674, 0.682, 0.700, and 0.782, respectively. This may be attributed to DSD’s disregard for relationships between and within feature classes, indicating that there is still room for improvement in grading accuracy. Moreover, DSD’s effectiveness depends heavily on data quality, which explains its poor performance in this study since our data are not preprocessed extensively. As shown in **Figure 5**, the DSD curve appears unstable, which suggests that DSD cannot be applied to our database.

Based on the analysis presented above, it can be concluded that the proposed featured selection method dl-LASSO can outperform the other five existing methods. LASSO adopts a shrinking (regularization) process to penalize the coefficients. Through the process of shrinking and removing coefficients, LASSO can reduce variance without causing significant bias. This makes it particularly effective in high-dimensional feature spaces, resulting in highly accurate feature selection. Furthermore, the proposed dl-LASSO method improves noise resistance and grading accuracy by considering the correlation between features and grading results as well as the relationships within and between features.

This study aims to address radiomics feature selection problems, and therefore, develops an efficient method for extracting discriminative features. Nonetheless, this work also has some limitations that remain critical roadblocks for its practical implementation in the HCC grading system. Firstly, the data only includes MR images of HCC from one hospital, which may lack diversity. It would be intriguing to explore the potential benefits of incorporating data from different imaging modalities (such as CT in addition to MR), different stages of the disease (including healthy data), and different detection methods, which may help to address the diversity limitation of the current study. In fact, we are in the process of doing this, we are now collecting data from different hospitals using CT or MR with different parameter settings. In our future work, therefore, we plan to investigate the performance of the proposed approach on a more comprehensive dataset. Secondly, the feature extraction process requires manual delineation of the tumor on multiple image slices. This process was time-consuming, and the segmentation performance relies on the experience of radiologists. The rapid progress in the field of deep learning has resulted in the emergence of automated segmentation of medical images, which could be potentially used in future studies. Finally, as this was a retrospective study, there is a possibility of bias in patient selection.

## 6 Conclusion

This study proposed a feature selection method based on LASSO with dictionary learning. Firstly, according to the influence of each feature on the grading result and the value of the vector *α*, each feature is divided into the high information part and the low information part. Subsequently, through the mapping matrix, the high-information part of the dictionary is strengthened and the low-information part is suppressed. Finally, the effectiveness of the proposed method has been assessed by a series of comparison experiments based on the grading performance. The experimental results based on two classifiers showed that the proposed method yielded accuracy gains, compared favorably with another 5 state-of-the-practice feature selection methods.

## Data availability statement

The raw data supporting the conclusions of this article will be made available by the authors, without undue reservation.

## Author contributions

Conceptualization: LL and Y-LH; Data acquisition and processing: L-XD, J-PY, and PW; Formal analysis: LL, Y-LH, and ZH; Investigation: LL, L-XD, and CW; Methodology: LL, Y-LH, and CW; Software: LL and Y-LH; Validation: LL and CW; Writing – original draft: LL; Writing – review and editing: L-XD, Y-LH, JY, PW, B-LY, CW, and ZH. All authors contributed to the article and approved the submitted version.
